# A Nature-Inspired Design Yields a New Class of Steroids Against Trypanosomatids

**DOI:** 10.3390/molecules24203800

**Published:** 2019-10-22

**Authors:** Elena Aguilera, Cintya Perdomo, Alejandra Espindola, Ileana Corvo, Paula Faral-Tello, Carlos Robello, Elva Serna, Fátima Benítez, Rocío Riveros, Susana Torres, Ninfa I. Vera de Bilbao, Gloria Yaluff, Guzmán Alvarez

**Affiliations:** 1 Grupo de Química Medicinal-Laboratorio de Química Orgánica, Facultad de Ciencias, Universidad de la República, Montevideo C.P. 11400, Uruguay; elepao168@gmail.com; 2 Laboratorio de Moléculas Bioactivas, CENUR Litoral Norte, Universidad de la República, Ruta 3 (km 363), Paysandú C.P. 60000, Uruguay; cuquis266@gmail.com (C.P.);; 3 Unidad de Biología Molecular, Institut Pasteur de Montevideo, Montevideo C.P. 11400, Uruguay; 4 Departamento de Bioquímica, Facultad de Medicina, Universidad de la República, Montevideo 11200, Uruguay; 5 Departamento de Medicina Tropical, Instituto de Investigaciones en Ciencias de la Salud, Universidad Nacional de Asunción, San Lorenzo C.P. 2169., Paraguay; elvsern@gmail.com (E.S.); fatimaapodaca14@gmail.com (F.B.); r.riverosmaidana@gmail.com (R.R.); susitorres1@hotmail.com (S.T.); nverabilbao@gmail.com (N.I.V.d.B.); 6 Facultad de Ciencias Exactas y Naturales, Universidad Nacional de Asunción, San Lorenzo C.P. 2169., Paraguay

**Keywords:** anti-*T. cruzi* activity in vitro and in vivo, anti-*Leishmaniaspp*. activity in vitro and in vivo, cytotoxicity and genotoxicity

## Abstract

Chagas disease and Leishmaniasis are neglected endemic protozoan diseases recognized as public health problems by the World Health Organization. These diseases affect millions of people around the world however, efficient and low-cost treatments are not available. Different steroid molecules with antimicrobial and antiparasitic activity were isolated from diverse organisms (ticks, plants, fungi). These molecules have complex structures that make de novo synthesis extremely difficult. In this work, we designed new and simpler compounds with antiparasitic potential inspired in natural steroids and synthesized a series of nineteen steroidal arylideneketones and thiazolidenehydrazines. We explored their biological activity against *Leishmania infantum*, *Leishmania amazonensis,* and *Trypanosoma cruzi* in vitro and in vivo. We also assayed their genotoxicity and acute toxicity in vitro and in mice. The best compound, a steroidal thiosemicarbazone compound **8** (ID_**1260)** was active in vitro (IC_50_ 200 nM) and in vivo (60% infection reduction at 50 mg/kg) in *Leishmania* and *T. cruzi*. It also has low toxicity in vitro and in vivo (LD_50_ >2000 mg/kg) and no genotoxic effects, being a promising compound for anti-trypanosomatid drug development.

## 1. Introduction

Chagas disease and Leishmaniasis are neglected endemic protozoan diseases recognized as public health problems by the World Health Organization [1]. Chagas disease, caused by the parasite *Trypanosoma cruzi*, is found mainly in Latin America and is estimated to affect 6–7 million people and at least 90–100 million people are at risk of infection in endemic areas [2]. Important advances in vector and transfusion controls of Chagas disease have been achieved by the Chagas Control Initiatives in the Americas; however, there is still a large number of chronic patients for whom treatment is not accessible or effective [3]. Leishmaniasis affects 12 million people in 98 countries, and a billion people are at risk of infection for being exposed to the parasite [3]. There are four clinical forms of the disease caused by more than 20 *Leishmania* species: Visceral Leishmaniasis (VL) (also called Kala-azar), dermal Leishmaniasis (post-kala-azar), Cutaneous Leishmaniasis (CL), and Mucocutaneous Leishmaniasis (MCL). The most common form is CL, however, VL can be fatal if untreated [4].

It is important to note that no vaccines are available for any of these diseases to prevent human infection and the drugs currently used for their treatment have high toxicity [5]. Benznidazole (commercial names Radanil^®^ Abarax^®^ or Rochagan^®^) and Nifurtimox (Lampit^®^) are highly effective for Chagas disease treatment during the acute phase and in congenital cases, which represent between 5% and 10% of the cases; however, the efficacy of these drugs decreases during the chronic phase [6]. The drugs currently used for the treatment of CL and VL have quite serious adverse reactions, like teratogenic and cardiotoxic effects. For the treatment of CL, parenteral administration of pentavalent antimony (sodium stibogluconate or pentostam^®^ antimoniate and meglumine or glucantime^®^) is carried out for 20 days; and in the case of VL, for 28 days. This must be done under supervised medical control, greatly increasing its cost. An alarming resistance against these drugs has been observed, while other drugs, such as pentamidine, rifampicin, amphotericin B, allopurinol, Miltefosine, and ketoconazole, also used to treat Leishmaniasis have limited therapeutic efficacy and also resistance problems [7]. It is then an urgent need to develop new, accessible, and effective drugs to control these diseases [7,8].

**Figure 1:**
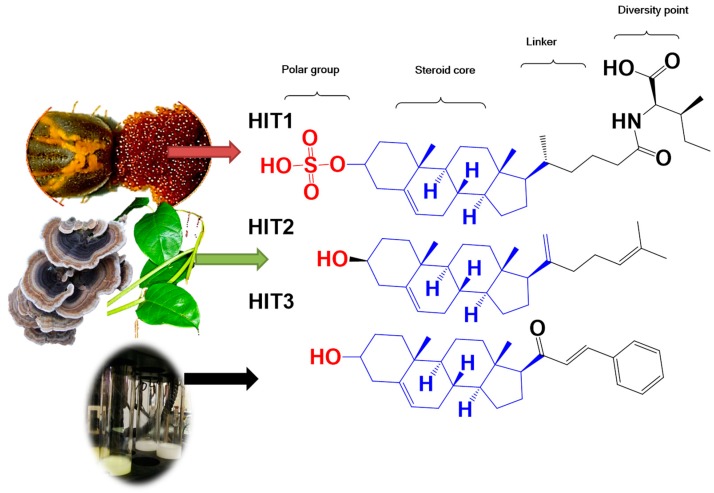
Boophiline (**HIT1**) is a steroid molecule present in *R. microplus* eggs that protects them against microbes. A similar steroid was reported with moderate antiparasitic activity from *P. andrieuxii* roots and *T. versicolor* (**HIT2**). In addition, **HIT3** was previously reported as a simple steroid compound with diverse biological activities [18,19]. Brackets show where structural modifications were made for the design of new steroid derivatives.

A steroid molecule isolated from the eggshell of the tick *Rhipicephalus microplus*, **HIT1** (*N*-(3-sulfoxy-25-cholest-5-en-26-oil)-*L*-isoleucine) was identified as an antimicrobial agent. This compound is a cholesterol sulfate attached to a residue of L-isoleucine via an amide bond (Figure 1) [9]. **HIT2** (cholest-4,20,24-trien-3-one), a cholesterol derivative extracted from *Pentalinon andrieuxii* roots exhibited potent activity against *Leishmania mexicana* promastigote and amastigote forms. In addition, a similar compound from *Trametes versicolor* was characterized with anti-Leishmania activity with an IC_50_ in *L. mexicana* amastigotes of 15 µM [10,11] Furthermore, steroid molecules similar to the synthetic **HIT3** (Figure 1) were described with antibacterial, antifungal, antiparasitic, and anticarcinogenic activity [9,12,13,14]. These compounds are less toxic to mammalian cells than nitroaromatic compounds used as antiparasitic drugs. Steroid compounds have proven to be less likely to induce resistance in addition to its greater bioavailability due to their ability to traverse cell membranes [15,16,17]. However, no mechanism of action was proposed in any of the models described [18]. 

Many natural substances are potentially useful for the development of new antiparasitic drugs. Among these, sterols are an important and ubiquitous class of compounds that provide promising scaffolds to design and develop new drugs for the treatment of antiparasitic infections like Chagas disease and Leishmaniasis. However, their structural complexity is a concern for its synthetic production [10,20,21,22,23]. Here, we synthesized and chemically characterized a series of nineteen steroidal arylideneketones and thiazolidenehydrazines by simple synthesis procedures. We designed three groups of new compounds: (1) Arylideneketones as **HIT3** analogs, (2) thiosemicarbazide derivatives similar to **HIT2**, and (3) thiazolidenehydrazines derivatives as **HIT1** analogs. We explored their biological activity against *L. infantum*, *L. braziliensis,* and *T. cruzi* in vitro and in vivo. We also studied their genotoxicity and acute toxicity in vitro and in mice and we explored their pharmacokinetic profiles. 

## 2. Results and Discussion

### 2.1. Synthesis of Steroidal Arylideneketones and Thiazolidenehydrazines

**Figure 2:**
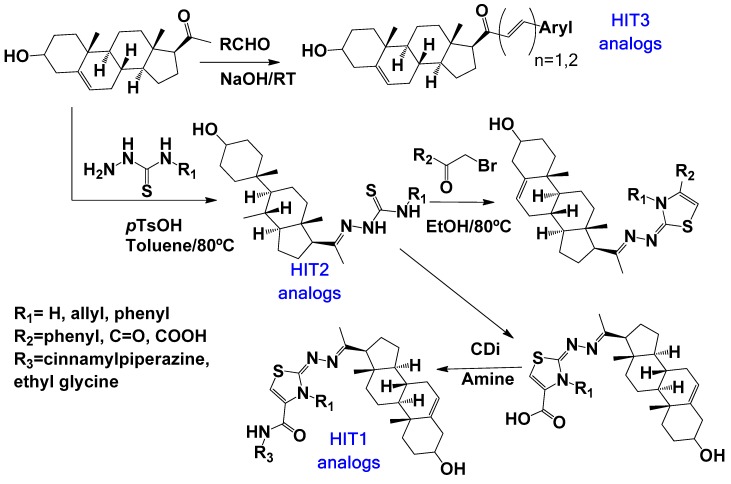
Synthetic procedure for steroidal arylideneketones and thiazolidenehydrazines. RT: Room temperature (25 °C), CDi: 1,1’-carbonyldiimidazole.

As part of our ongoing program in drug development for Chagas disease, we use simple reagents and simple conditions to get new molecules. The key intermediate was synthesized from commercially available pregnenolone using the same procedures previously reported (Figure 2) [24,25,26]. The stereochemistry of the compounds was determined by pregnenolone (3β-hydroxyl-5-pregnen-20-one, 5-pregnen-3β-ol-20-one). For compounds design we combined different features of **HIT1**, **2**, and **3** structures, using simple chemical procedures and low-cost reactants. 

We synthesized nineteen new compounds with good to excellent reaction yields. For the arylideneketones derivatives, we used a classical aldol reaction following the twelve green chemistry principles [27]. All of the new compounds were characterized by ^1^H-NMR, ^13^C-NMR, COSY, HSQC, HMBC experiments and Mass Spectrometry. The compounds, according to the H-H coupling constants and the NOE-diff experiments were obtained as the Z-isomers in the case of the thiazolidenehydrazines at the double bond generated from cyclization (thiazolidene ring). The purity of the synthesized compounds was assayed by TLC and LC-MS.

### 2.2. In Vitro Biological Studies

To evaluate their antiparasitic activity, we performed a phenotypic screening using the promastigote form of *L. infantum, L. braziliensis*, *L. amazonensis,* and the epimastigote form of *T. cruzi*, Tulahuen 2 strain (genotype TcVI) [28] (Table 1) at an initial concentration of 25 µM. We defined as active compounds those with IC_50_<25 µM. The solubility in water of the compounds was poor so they were tested at a maximum dose of 25 µM.

**Figure 3:**
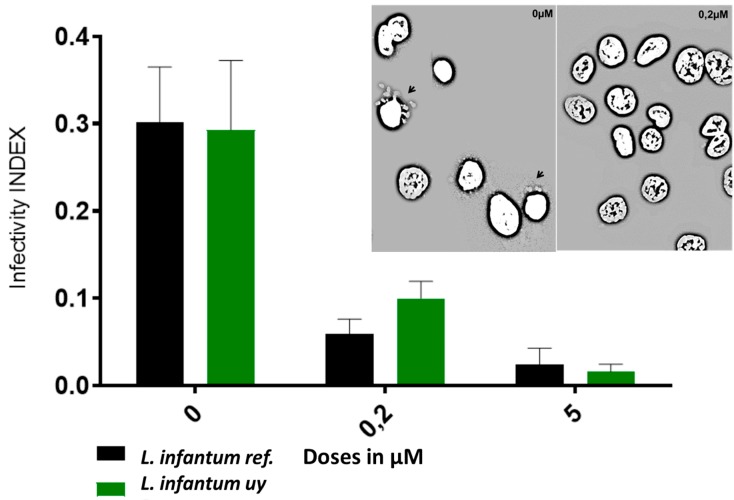
Effect of compound **8** at 200 nM and 5 µM over human macrophages infected with L. infantum. The infectivity index is the ratio between the number of amastigotes per cell before and after treatment. A fluorescence microscopy image with DAPI (2-(4-amidinophenyl)-1H -indole-6-carboxamidine) staining of human macrophages infected with *L. infantum* is shown (0 µM: Control and at the right side, cells treated with 0.2 µM of compound **8**).Amastigotes are shown with black arrows in the Control condition around the nucleus.

For the three compounds derived from the condensation of pregnenolone with thiosemicarbazides, all of the chemical motifs used yielded active molecules. In contrast, the arylideneketones derivatives did not show any activity at the evaluated doses. We showed a qualitative correlation between the *N* substituent in the thiosemicarbazides with the lipophilicity or size, because compounds with a phenyl group were less active than those with allyl substituent and no-substitute derivative consecutively. Compound **8** (ID_**1260**) was the most active compound in all species of parasites. In vitro it was 35 times more active than Nfx and Bnz, and five times more active than Miltefosine against *T. cruzi* and *Leishmania,* respectively. Moreover, this compound was active in a *Leishmania infantum* strain isolated from infected dogs in Uruguay [29] (Table 1). What is more, compound **8** showed an IC_50_ of less than 200 nM in human macrophages infected with two different strains of *L. infantum* (the reference strain and the isolated from dogs*),* being able to clean the amastigotes from the cell’s cytoplasm (Figure 3). This is in good correlation with the activity against promastigotes that we have also seen. Our results suggest the strong ability of this molecule to affect different forms and species of kinetoplastids. 

An interesting finding was that compounds **2**, **4**, **9**, and **14** were active against *Leishmania infantum* from Fiocruz collection but not when tested against the strains isolated from Uruguayan dogs. We need more studies in order to explain this differential behavior but it is important to bear in mind that the pharmacological response of a drug might differ between strains from different geographic regions. Only compound **8** was consistently active against all strains.

We evaluated the selectivity of the most active compounds by nonspecific cytotoxicity experiments against mammalian cells using J774.1 murine macrophages. The selectivity indexes were calculated as the ratio between IC_50_ for mammalian cells and IC_50_ for the parasites (Table 2). Compound **8** was the most selective compound, even if we compare it with their analogs **9** and **10.** However, these two molecules were poorly soluble in the conditions that mammalian cells were assayed, then the maximum dose tested was 25 µM and this influenced the low selectivity indexes we observed. We also studied the cytotoxicity of compound **8** using a primary culture of macrophages from mice bone marrow and the same results were obtained. 

We are the first to report this kind of biological activity for compound **8**. Its synthesis is a one-step reaction with excellent yields that can be done using green chemistry methods and requires low-cost reactants. In contrast, the amide synthesis (compound **12** for example) has more steps and expensive reactants. Then, compound **8** can be easily scaled up at low-cost and high yields, which makes it a promising drug for the treatment of neglected diseases.

### 2.3. Toxicology In Vivo

We chose compound **8** for toxicology in vivo assays because it was the most potent and selective in vitro. To evaluate genotoxicity we performed the micronucleus assay in mice (Table 3). The exposition of mice to a single oral dose of 150 mg/Kg of the compound did not induce chromatin damage on bone marrow cells, as the generation of multimicronucleated cells was similar to the negative control. 

**Figure 4:**
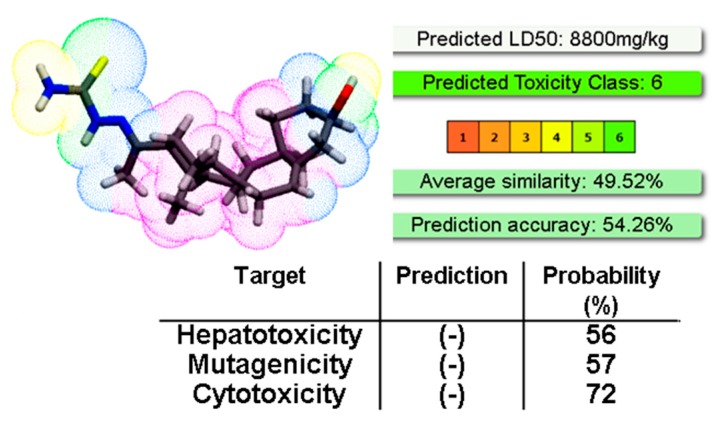
We show the output screen obtained from the open-access toxicity prediction software ProTox-II using compound **8**, (-) indicates no toxicity. The structural similarity of the compound with those in the software database is around 50%.

Interestingly, compound **8** has no acute toxicity in mice, as the LD_50_ estimated in an up and down experiment was >2000 mg/kg of body weight, the maximum dose that OECD guidelines recommend to test_._ In accordance, the in silico prediction of LC_50_ using the ProTox-II software was even higher (Figure 4). The software also predicted that compound **8** is not hepatotoxic, mutagenic, or cytotoxic (Figure 4).

### 2.4. Proof of Concept in Vivo

**Figure 5:**
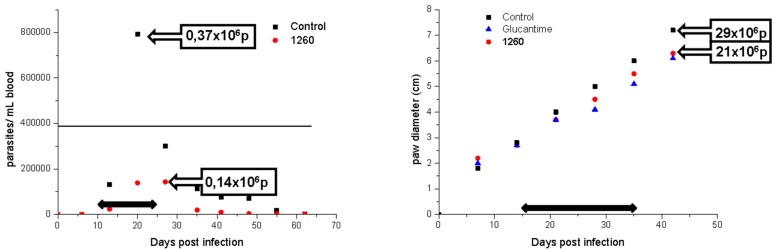
In vivo efficacy of compound **8** in mice models for Chagas disease (left) and Leishmaniasis (right). The black arrow indicates the treatment period where a dose of 50 mg/kg was administered once a day. In the left graph, the parasite number per mL of blood (parasitemia) is shown at different days post-infection. The black line indicates 50% of parasitemia relative to the control without treatment. In the right graph, the paw diameter is represented over time post-infection and the number of parasites in the lesion at the end of the experiment is indicated.

We evaluated compound **8** in an in vivo model of acute Chagas disease and Cutaneous Leishmaniasis (Figure 5). To compare the efficacy of our compound we incorporated the reference drugs Benznidazole for Chagas disease and Glucantime for Leishmaniasis. We chose oral administration because it is the desirable route of administration for vulnerable populations where these neglected diseases are more frequent. As compound **8** showed poor solubility in water, we developed a new vehicle to favor the solubilization of lipophilic drugs to increase gastrointestinal absorption (see details in the experimental section).

The in vivo efficacy in both models was similar or better than the reference drugs (Figure 5 and Table 4). For the cutaneous model of Leishmaniasis, compound **8** was better than Glucantime at less than half the dose (127 compared to 273 µmol/kg). A similar result was obtained in the acute model of Chagas disease, where our compound showed 62% reduction of parasitemia compared to 96% with Benznidazole, but administering almost half the dose (127 compared to 200 µmol/kg). 

Finally, we performed metabolic stability studies using microsomal and cytosolic hepatic fractions and used the open-access software to predict pharmacokinetic parameters (Table 5). Compound **8** was stable in a cytosolic and microsomal liver fraction for 4 h (supporting information). Moreover, the pharmacokinetic parameters are similar or even better than those of available drugs as seen for Miltefosine and Glucantime [30,31]. 

## 3. Experimental Section

### 3.1. General

Reagents were purchased from Aldrich and used without further purification. Melting points were performed using an Electrothermal Engineering Ltd. melting point apparatus, and the results were not corrected. ^1^H NMR and ^13^C NMR spectra were recorded in the indicated solvent with a Bruker DPX 400-MHz spectrometer. Chemical shifts are quoted in parts per million downfield from tetramethylsilane (TMS), and the coupling constants are in Hertz. Structural assignments were corroborated by COSY, HMBC, and HSQC experiments. All solvents were dried and distilled prior to use. All of the reactions were carried out in a nitrogen atmosphere. Reactions were monitored by thin-layer chromatography (TLC) using commercially available precoated plates (Merck Kieselgel 60 F254 silica), and the developed plates were examined under UV light (254 nm) or as iodine vapor stains. Column chromatography was performed using a 200 mesh silica gel. Mass spectrometry experiments were performed on a HEWLETT PACKARD MSD 5973 or an LC/MSD-Serie 100 using electronic impact (EI) or electrospray ionization (ESI), respectively. To determine the purity of the compounds, microanalyses were done on a Fisons EA 1108 CHNS–O instrument from vacuum-dried samples and were within ± 0.4 of the values obtained by calculating their compositions. The compounds were prepared following synthetic procedures previously reported [12,15,16,17].

#### 3.1.1. General Synthetic-Procedure for Arylidene Ketones

To a solution of pregnenolone (0.316 g, 1.0 equivalent (eq.)) in ethanol (10 mL) was added a concentrated aquoeus solution of KOH (2.0 eq.). Then, the corresponding aldehyde (1.2 eq.) was added into the reaction mixture to get the corresponding benzylidine derivative. After completion, as revealed by the thin-layer chromatography (TLC) in an average span of around 1 h, the reaction mixture was precipitated using water because of the limited solubility. The precipitate was filtered, dried and purity was monitored through TLC. It revealed just a single spot which proved the presence of a single product. For further purification, the product was recrystallized from EtOH to obtain it as a solid [13].


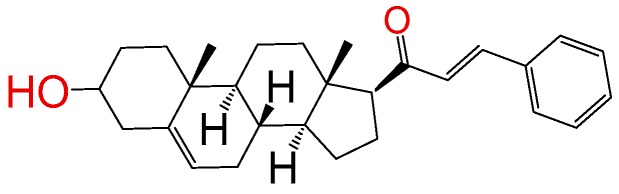


##### Compound **5**

(2*E*)-1-((10*R*,13*S*)-2,3,4,7,8,9,10,11,12,13,14,15,16,17-tetradecahydro-3(*β*)-hydroxy-10,13-dimethyl-1*H*-cyclopenta[a]phenan-thren-17(*β*)-yl)-3-phenylprop-2-en-1-one (**1288**).[13] Yield: 86%, white powder, mp: 128–131 °C; ^1^H RMN (400 MHz, CDCl_3_) δ (ppm): 0.63 (s, 3H), 1.00 (s, 3H), 1.61–1.90 (m, 6H), 2.20–2.38 (m, 3H), 2.82 (t, *J* = 8.80, 1H); 3.51 (m, 1H); 6.78 (d, *J* = 16.0 Hz, 1H), 7.39 (m, 3H), 7.55 (m, 3H); ^13^C RMN (100 MHz, CDCl_3_) δ (ppm): 13.3, 19.5, 21.4, 23.9, 25.4, 31.7, 32.2, 32.8, 37.4, 42.1, 45.7, 49.6, 49.2, 49.6, 49.9, 49.9, 50.5, 57.4, 62.3, 71.4, 121.2, 127.7, 128.4, 129.5, 130.2, 135.3, 141.3, 142.4, 201.7 C_28_H_35_O_2_. ESI-MS (m/z): 404.27 (100.0%), 405.27 (30.3%), 406.28 (5.0%). Elemental analysis: C, 83.12; H, 8.97; O, 7.91.


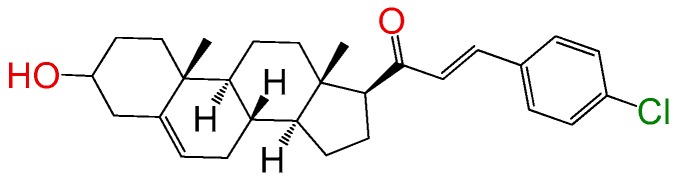


##### Compound **3**

(2*E*)-3-(4-Chlorophenyl)-1-((10*R*,13*S*)-2,3,4,7,8,9,10,11,12,13,14,15,16,17-tetradecahydro-3(*β*)-hydroxy-10,13-dimethyl-1*H*-cyclo- penta[a]phenanthren-17(*β*)-yl)prop-2-en-1-one (**1256**). Yield: 85%, white powder, mp: 159–161 °C; ^1^H RMN (400 MHz CDCl_3_) δ (ppm): 0.63 (s, 3H), 0.99 (s, 3H), 2.83 (t, 1H, *J* = 8.5 Hz), 3.52 (m, 1H), 5.35 (t, 1H, *J* = 2.5 Hz), 6.73 (d, 1H, *J* = 16.0 Hz), 7.35 y 7.47 (d, 2H, *J* = 8.0 Hz, y d, 2H, *J* = 8.0 Hz), 7.49 (d, 1H, *J* = 16.0 Hz). ^13^C RMN (100 MHz CDCl_3_) δ (ppm): 13.2, 19.5, 21.7, 23.4, 25.4, 31.7, 32.3, 32.8, 37.4, 37.9, 39.2, 42.2, 45.4, 50.5, 57.3, 62.4, 71.7, 121.2, 127.3, 129.3, 129.4, 133.7, 136.1, 140.1, 141.5, 200.2 C_28_H_35_ClO_2_. ESI-MS (m/z): 438.23 (100.0%), 440.23 (32.0%), 439.24 (30.8%), 441.23 (9.7%), 440.24 (5.0%), 442.24 (1.5%). Elemental analysis: C, 76.60; H, 8.04; Cl, 8.08; O, 7.29.


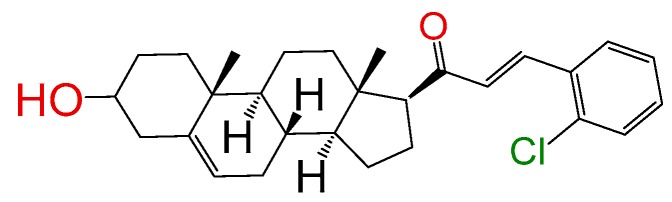


##### Compound **2**

2*E*)-3-(2-Chlorophenyl)-1-((10*R*,13*S*)-2,3,4,7,8,9,10,11,12,13,14,15,16,17-tetradecahydro-3(*β*)-hydroxy-10,13-dimethyl-1*H*-cyclo-penta[a]phenanthren-17(*β*)-yl)prop-2-en-1-one (**1259**). Yield: 86% white powder, mp 149–151 °C; ^1^H RMN (400 MHz CDCl_3_) δ (ppm): 0.63 (s, 3H), 0.99 (s, 3H), 2.83 (t, 1H, J = 8.5 Hz), 3.52 (m, 1H), 5.35 (t, 1H, J = 2.5 Hz), 6.73 (d, 1H, J = 16.0 Hz), 7.49 (m, 4H). ^13^C RMN (100 MHz CDCl_3_) δ (ppm): 13.3, 19.3, 21.4, 23.6, 25.2, 32.3, 32.5, 32.9, 36.2, 37.2, 39.1, 42.7, 45.9, 50.4, 57.5, 62.3, 72.4, 121.2, 127.4, 129.4, 129.4, 133.2, 136.7, 140.4, 141.4, 200.2 C_28_H_35_ClO_2_. ESI-MS (m/z): 438.23 (100.0%), 440.32 (31.0%), 439.21 (29.8%), 441.50 (8.7%), 440.24 (5.0%), 442.24 (1.5%). Elemental analysis: C, 76.60; H, 8.04; Cl, 8.08; O, 7.29.


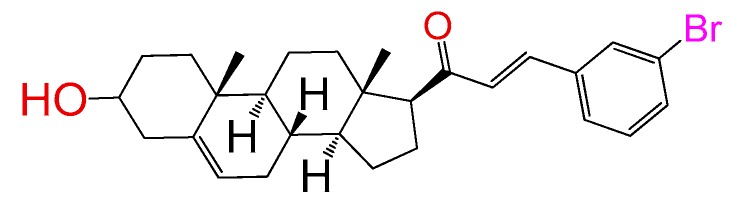


##### Compound **4**

2*E*)-3-(3-bromophenyl)-1-((10*R*,13*S*)-2,3,4,7,8,9,10,11,12,13,14,15,16,17-tetradecahydro-3(*β*)-hydroxy-10,13-dimethyl-1*H*-cyclo-penta[a]phenanthren-17(*β*)-yl)prop-2-en-1-one (**1417**). Yield: 96% white powder, mp 159–161 °C; ^1^H RMN (400 MHz CDCl_3_) δ (ppm): 0.63 (s, 3H), 0.99 (s, 3H), 2.83 (t, 1H, J = 8.5 Hz), 3.52 (m, 1H), 5.35 (t, 1H, J = 2.5 Hz), 6.73 (d, 1H, J = 16.0 Hz), 7.49 (m, 3H), 7.52 (s, 1H). ^13^C RMN (100 MHz CDCl_3_) δ (ppm): 13.3, 19.3, 21.4, 23.6, 25.2, 32.3, 32.5, 32.9, 36.2, 37.2, 39.1, 42.7, 45.9, 50.4, 57.5, 62.3, 72.4, 121.2, 128.4, 130.4, 130.8, 136.2, 140.7, 143.4, 144.4, 200.2 C_28_H_35_BrO_2_. ESI-MS (m/z): 482.18 (100.0%), 484.18 (97.3%), 483.19 (30.8%), 485.18 (29.5%), 486.19 (4.5%). Elemental analysis: C, 69.56; H, 7.30; Br, 16.53; O, 6.62.


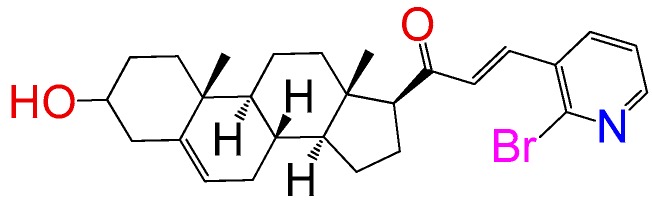


##### Compound **1**

2*E*)-3-(2-bromopyridin-3-yl)-1-((10*R*,13*S*)-2,3,4,7,8,9,10,11,12,13,14,15,16,17-tetradecahydro-3(*β*)-hydroxy-10,13-dimethyl-1*H*-cyclo-penta[a]phenanthren-17(*β*)-yl)prop-2-en-1-one (**1257**). Yield: 86% white powder, mp 193–195 °C; ^1^H RMN (400 MHz CDCl_3_) δ (ppm): 0.63 (s, 3H), 0.99 (s, 3H), 2.83 (t, 1H, J = 8.5 Hz), 3.52 (m, 1H), 5.35 (t, 1H, J = 2.5 Hz), 6.73 (d, 1H, J = 16.0 Hz) 7.5 (t, 1H, J = 8.0 Hz), 7.8 (d, 1H, J = 8.0 Hz), 8.0 (d, 1H, J = 8.0 Hz). ^13^C RMN (100 MHz CDCl3) δ (ppm): 13.3, 19.4, 21.2, 22.8, 24.3, 31.4, 31.8, 32.2, 36.4, 37.7, 39.5, 42.1, 45., 50.2, 57.4, 62.4, 72.4, 121.7, 123.5, 132.7, 136.6, 139.4, 140.5, 142.4, 148.7, 204.2 C_27_H_34_BrNO_2_. ESI-MS (m/z): 485.18 (100.0%), 483.18 (98.1%), 484.18 (29.1%), 486.18 (28.5%), 487.18 (4.5%). Elemental analysis: C, 66.94; H, 7.07; Br, 16.49; N, 2.89; O, 6.60.


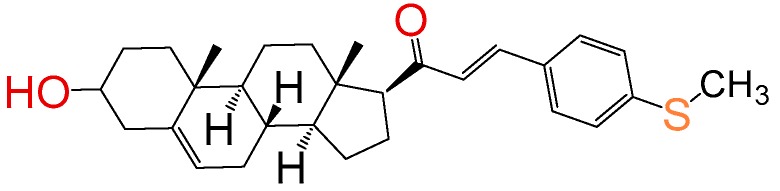


##### Compound **15**

2*E*)-3-(4-(methylthio)phenyl)-1-((10*R*,13*S*)-2,3,4,7,8,9,10,11,12,13,14,15,16,17-tetradecahydro-3(*β*)-hydroxy-10,13-dimethyl-1*H*-cyclo-penta[a]phenanthren-17(*β*)-yl)prop-2-en-1-one (**1262**). Yield: 98% yellow powder, mp 124–126 °C; ^1^H RMN (400 MHz CDCl_3_) δ (ppm): 0.63 (s, 3H), 0.99 (s, 3H), 2.53 (s, 3H), 2.83 (t, 1H, J = 8.5 Hz), 3.52 (m, 1H), 5.35 (t, 1H, J = 2.5 Hz), 6.73 (d, 1H, J = 16.0 Hz), 7.35 y 7.47 (d, 2H, J = 8.0 Hz, y d, 2H, J = 8.0 Hz), 7.49 (d, 1H, J = 16.0 Hz). ^13^C RMN (100 MHz CDCl_3_) δ (ppm): 13.2, 15.4, 19.3, 21.1, 23.2, 25.4, 32.2, 32.6, 32.9, 37.2, 37.3, 39.8, 42.1, 45.4, 50.1, 57.5, 62.1, 71.2, 121.2, 127.8, 129.4, 129.8, 133.2, 136.5, 140.1, 140.5, 200.3 C_29_H_38_O_2_S. ESI-MS (m/z): 450.26 (100.0%), 451.26 (32.2%), 452.26 (10.2%), 453.26 (1.4%). Elemental analysis: C, 77.29; H, 8.50; O, 7.10; S, 7.11.


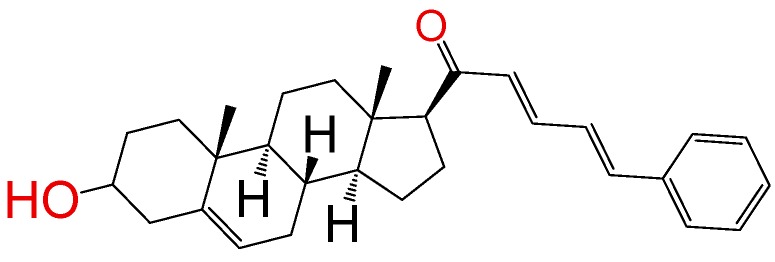


##### Compound **6**

(2*E*,4*E*)-1-((*8S*,9*S*,10*R*,13*S*,14*S*,17*S*)-3-hydroxy-10,13-dimethyl-2,3,4,7,8,9,10,11,12,13,14,15,16,17-tetradecahydro-1*H*-cyclopenta[a]phenanthren-17-yl)-5-phenylpenta-2,4-dien-1-one (**1279**). Yield: 99% yellow powder, mp 116–119 °C; ^1^H RMN (400 MHz, CDCl_3_) δ (ppm): 0.63 (s, 3H), 1.00 (s, 3H), 1.61–1.90 (m, 6H), 2.20–2.38 (m, 3H), 2.82 (t, *J* = 8.80, 1H); 3.51 (m, 1H); 6.78 (d, *J* = 16.0 Hz, 1H), 6.73 (t, 1H, *J* = 11.0 Hz), 7.39 (m, 3H), 7.55 (m, 3H), 7.49 (t, 1H, *J* = 11.0 Hz). ^13^C RMN (100 MHz, CDCl_3_) δ (ppm): 13.1, 19.2, 21.3, 22.1, 24.3, 31.0, 31.5, 31.9, 37.2, 41.2, 45.4, 48.1, 48.5, 48.7, 49.2, 49.6, 50.1, 57.5, 62.2, 71.2, 121.2, 125.5, 126.5, 128.7, 129.5, 130.2, 134.3, 140.2, 141.6, 142.2, 201.3 C_30_H_38_O_2_. ESI-MS (m/z): 430.29 (100.0%), 431.29 (33.0%), 432.29 (5.5%). Elemental analysis: C, 83.67; H, 8.89; O, 7.43.


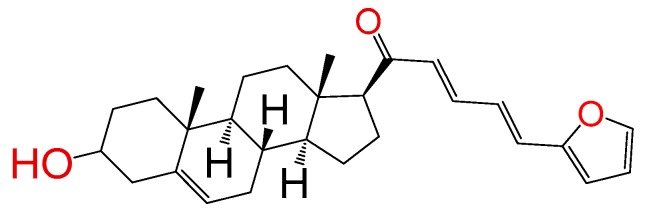


##### Compound **7**

(2*E*,4*E*)-5-(furan-2-yl)-1-((8*S*,9*S*,10*R*,13*S*,14*S*,17*S*)-3-hydroxy-10,13-dimethyl-2,3,4,7,8,9,10,11,12,13,14,15,16,17-tetradecahydro-1*H*-cyclopenta[a]phenanthren-17-yl)penta-2,4-dien-1-one (**1289**). Yield: 98% yellow powder, mp 100–103 °C; ^1^H RMN (400 MHz, CDCl_3_) δ (ppm): 0.63 (s, 3H), 1.00 (s, 3H), 1.61–1.90 (m, 6H), 2.20–2.38 (m, 3H), 2.82 (t, *J* = 8.80, 1H); 3.51 (m, 1H); 6.33 (d, *J* = 16.0 Hz, 1H), 6.52 (m, 1H), 6.65 (d, *J* = 16.0 Hz, 1H), 6.71 (m, 1H), 6.75 (m, 1H), 6.78 (m, 1H), 6.95 (d, *J* = 8 Hz, 1H), 7.45 (m, 1H), 7.75 (d, J = 8, 1H). ^13^C RMN (100 MHz, CDCl_3_) δ (ppm): 13.3, 19.2, 21.2, 23.2, 25.4, 31.7, 32.2, 32.5, 37.5, 42.2, 45.4, 49.2, 49.5, 49.8, 49.9, 50.1, 50.5, 57.2, 62.5, 71.4, 109.4, 109.5, 112.4, 121.7, 130.4, 139.2, 140., 144.5, 147.4, 151.2, 200.3 C_28_H_36_O_3_ ESI-MS (m/z): 420.27 (100.0%), 421.27 (30.8%), 422.27 (5.1%). Elemental analysis: C, 79.96; H, 8.63; O, 11.41.


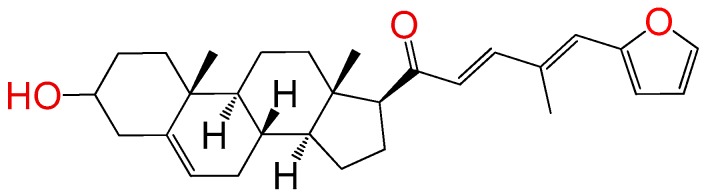


##### Compound **16**

(2*E*,4*E*)-5-(furan-2-yl)-1-((8*S*,9*S*,10*R*,13*S*,14*S*,17*S*)-3-hydroxy-10,13-dimethyl-2,3,4,7,8,9,10,11,12,13,14,15,16,17-tetradecahydro-1*H*-cyclopenta[a]phenanthren-17-yl)-4-methylpenta-2,4-dien-1-one (**1287**). Yield: 96% yellow powder, mp: 110–113 °C; ^1^H RMN (400 MHz, CDCl_3_) δ (ppm): 0.63 (s, 3H), 1.00 (s, 3H), 1.61–1.90 (m, 6H), 2.20–2.38 (m, 6H), 2.82 (t, *J* = 8.80, 1H); 3.51 (m, 1H); 6.33 (d, *J* = 16.0 Hz, 1H), 6.52 (m, 1H), 6.65 (d, *J* = 16.0 Hz, 1H), 6.75 (m, 1H), 6.78 (m, 1H), 6.95 (d, *J* = 8 Hz, 1H), 7.45 (m, 1H), 7.75 (d, *J* = 8, 1H). ^13^C RMN (100 MHz, CDCl_3_) δ (ppm): 13.5, 14.2, 19.1, 21.0, 23.2, 25.4, 31.3, 32.3, 32.9, 37.2, 42.1, 45.4, 49.1, 49.4, 49.7, 49.9, 50.1, 50.5, 57.2, 62.1, 71.0,109.4, 109.8, 112.2, 121.5, 130.5, 139.8, 140.2, 144.3, 147.6, 151.2, 200.3 C_29_H_38_O_3_ ESI-MS (m/z): 434.28 (100.0%), 435.29 (31.9%), 436.29 (5.5%). Elemental analysis: C, 80.14; H, 8.81; O, 11.04.


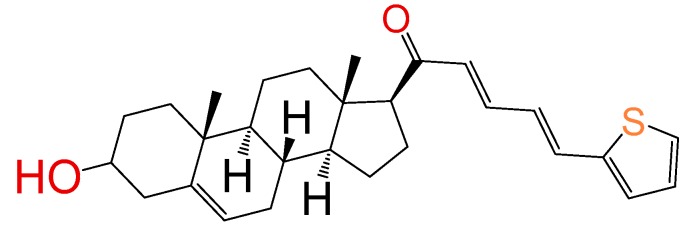


##### Compound **17**

(2*E*,4*E*)-5-(thiofen-2-yl)-1-((8*S*,9*S*,10*R*,13*S*,14*S*,17*S*)-3-hydroxy-10,13-dimethyl-2,3,4,7,8,9,10,11,12,13,14,15,16,17-tetradecahydro-1*H*-cyclopenta[a]phenanthren-17-yl)penta-2,4-dien-1-one (**1319**). Yield: 53% yellow powder, mp 158–164 °C; ^1^H RMN (400 MHz, CDCl_3_) δ (ppm): 0.63 (s, 3H), 1.00 (s, 3H), 1.61–1.90 (m, 6H), 2.20–2.38 (m, 3H), 2.82 (t, *J* = 8.80, 1H); 3.51 (m, 1H); 6.33 (d, *J* = 16.0 Hz, 1H), 6.52 (m, 1H), 6.65 (d, *J* = 16.0 Hz, 1H), 6.71 (m, 1H), 6.75 (m, 1H), 6.78 (m, 1H), 7.04 (d, *J* = 8 Hz, 1H), 7.17 (m, 1H), 7.69 (d, *J* = 8, 1H). ^13^C RMN (100 MHz, CDCl_3_) δ (ppm): 13.2, 19.3, 21.2, 23.2, 25.4, 31.3, 32.1, 32.5, 37.6, 42.5, 45.5, 49.2, 49.4, 49.6, 49.8, 49.9, 50.2, 57.5, 62.2, 71.2, 121.3, 121.5, 128.2, 129.5, 130.5, 139.4, 140.5, 144.7, 147.9, 151.5, 200.2 C_30_H_38_O_2_S. ESI-MS (m/z): 436.24 (100.0%), 437.25 (30.8%), 438.25 (9.7%), 439.24 (1.4%). Elemental analysis: C, 77.02; H, 8.31; O, 7.33; S, 7.34.

#### 3.1.2. General Synthetic-Procedure for Thiosemicarbazones

A mixture of pregnenolone (1.0 eq.), the corresponding thiosemicarbazides (1.0 eq.), catalytic amount of p-toluenesulfonic acid, and dry toluene (1 mL per 100 mg of reactants) was stirred at 80 °C until disappearance of the pregnenolone (5–8 h, checked by TLC, SiO_2_, and petroleum ether/EtOAc 70:30). After that, the precipitate was filtered off and washed with petroleum ether. The solid was crystallized from ethanol [16,17,25,32].


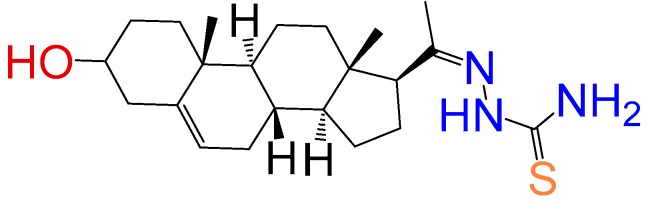


##### Compound **8**

1-(3*β*-Hydroxy-pregn-5-ene-20*E*-ylidene)thiosemicarbazone (**1260**). Yield: 95%, white powder, mp: 245–248 °C; ^1^H RMN (400 MHz CDCl_3_) δ (ppm): 0.63 (s, 3H), 0.99 (s, 3H), 2.83 (t, 1H, *J* = 8.5 Hz), 3.52 (m, 1H), 5.35 (t, 1H, *J* = 2.5 Hz), 8.56 (s, 2H). ^13^C RMN (100 MHz CDCl_3_) δ (ppm): 13.2, 19.5, 21.2, 23.5, 25.2, 31.4, 32.6, 32.9, 37.2, 37.8, 39.5, 42.5, 45.5, 50.7, 57.5, 62.4, 71.2, 121.4, 165.4, 181.2 C_22_H_35_N_3_OS. ESI-MS (m/z): 389.25 (100.0%), 390.25 (25.7%), 391.25 (10.0%), 392.25 (1.1%). Elemental analysis: C, 67.82; H, 9.05; N, 10.79; O, 4.11; S, 8.23.


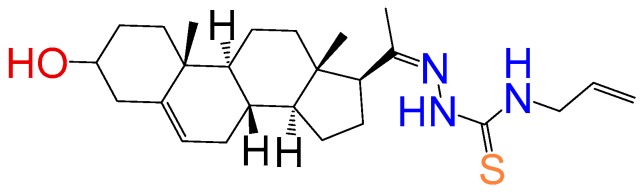


##### Compound **9**

1-(3*β*-Hydroxy-pregn-5-ene-20*E*-ylidene)-4-Allylthiosemicarbazone (**1154**). Yield 95%, white powder, mp 229–233 °C; ^1^H RMN (400 MHz CDCl_3_) δ (ppm): 0.63 (s, 3H), 0.99 (s, 3H), 2.83 (t, 1H, *J* = 8.5 Hz), 3.52 (m, 1H), 3.35 (m, 1H), 5.21 (d, 1H, *J* = 16 Hz), 5.32 (d, 1H, *J* = 10 Hz), 5.35 (m, 2H), 5.77 (m, 1H), 8.56 (s, 1H). ^13^C RMN (100 MHz CDCl_3_) δ (ppm): 13.2, 19.3, 21.5, 23.4, 25.8, 31.9, 32.2, 32.5, 37.8, 37.4, 39.7, 42.3, 45.5, 47.8, 50.2, 57.6, 62.3, 71.2, 117.6, 121.2, 134.5, 165.6, 181.2. C_25_H_39_N_3_OS. ESI-MS (m/z): 429.28 (100.0%), 430.28 (28.9%), 431.28 (5.1%), 431.29 (3.9%), 432.28 (1.2%). Elemental analysis: C, 69.88; H, 9.15; N, 9.78; O, 3.72; S, 7.46.


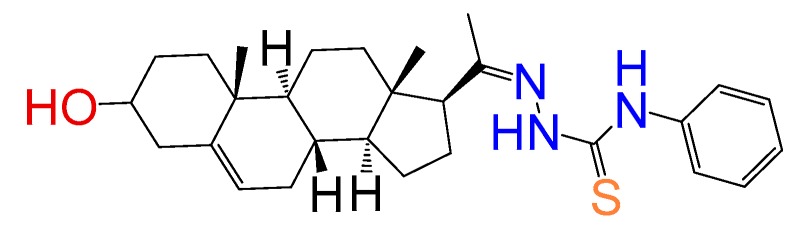


##### Compound **10**

1-(3*β*-Hydroxy-pregn-5-ene-20*E*-ylidene)-4-phenylthiosemicarbazone (**1291**).[17] Yield 19% white powder, mp: 215–217 °C; ^1^H RMN (400 MHz CDCl3) δ (ppm): 0.63 (s, 3H), 0.99 (s, 3H), 2.83 (t, 1H, *J* = 8.5 Hz), 3.52 (m, 1H), 3.35 (m, 1H), 6.81 (m, 1H), 7.20 (m, 2H), 7.7 (d, 2H, *J* = 8.5 Hz). ^13^C RMN (100 MHz CDCl_3_) δ (ppm): 13.2, 19.3, 21.5, 23.7, 25.8, 31.8, 32.2, 32.9, 37.5, 37.5, 39.4, 42.4, 45.5, 50.5, 57.5, 62.2, 71.8, 121.7, 126.5, 128.7, 1298, 138.2, 165.5, 181.7 C_28_H_39_N_3_OS. ESI-MS (m/z): 465.28 (100.0%), 466.28 (32.2%), 467.28 (5.1%), 467.29 (4.8%), 468.28 (1.4%). Elemental Analysis: C, 72.21; H, 8.44; N, 9.02; O, 3.44; S, 6.89.

#### 3.1.3. General Synthetic-Procedure for Thiazolylidene Hydrazines 

A mixture of the corresponding thiosemicarbazone [25] (1.0 eq.), the α- haloketone (1.2 eq.), and dry ethanol (1 mL per 100 mg of thiosemicarbazone) was heated at reflux until the disappearance of the thiosemicarbazide (4–10 h, checked by TLC, SiO_2_, petroleum ether:EtOAc 70:30). After that, the mixture was cooled to room temperature, and the precipitate was filtered off and washed with ethanol: Water (80:20). The solid was crystallized from ethanol or ethanol: Water.


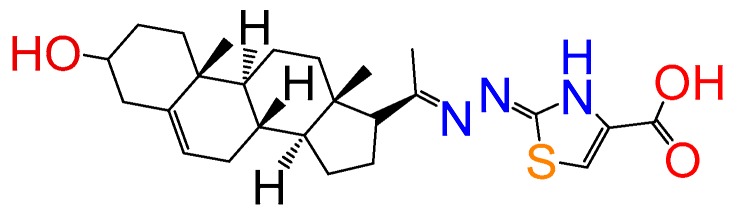


##### Compound **18**

(*Z*)-2-((*E*)-(1-((8*S*,9*S*,10*R*,13*S*,14*S*,17*R*)-3-hydroxy-10,13-dimethyl-2,3,4,7,8,9,10,11,12,13,14,15,16,17-tetradecahydro-1*H*-cyclopenta[a]phenanthren-17-yl)ethylidene)hydrazono)-2,3-dihydrothiazole-4-carboxylic acid (**1258**). Yield 99% yellow powder, mp: 281–283 °C; ^1^H RMN (400 MHz CDCl_3_) δ (ppm): 0.63 (s, 3H), 0.99 (s, 3H), 2.83 (t, 1H, J = 8.5 Hz), 3.52 (m, 1H), 5.35 (t, 1H, J = 2.5 Hz), 7.76 (s, 1H). ^13^C RMN (100 MHz CDCl_3_) δ (ppm): 13.2, 19.6, 21.4, 23.5, 25.5, 31.2, 32.2, 32.5, 37.4, 37.8, 39.2, 42.5, 45.5, 50.5, 57.5, 62.5, 71.2, 117.5, 121.5, 146.5, 146.5, 158.5, 163.5, 165.8, 180.2. C_25_H_35_N_3_O_3_S. ESI-MS (m/z): 457.24 (100.0%), 458.24 (29.1%), 459.24 (9.4%), 460.24 (1.3%). Elemental analysis: C, 65.61; H, 7.71; N, 9.18; O, 10.49; S, 7.01.


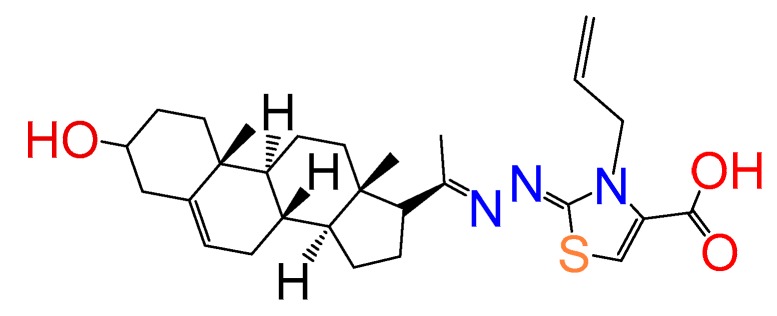


##### Compound **19**

(*Z*)-3-allyl-2-((*E*)-(1-((8*S*,9*S*,10*R*,13*S*,14*S*,17*R*)-3-hydroxy-10,13-dimethyl-2,3,4,7,8,9,10,11,12,13,14,15,16,17-tetradecahydro-1*H*-cyclopenta[a]phenanthren-17-yl)ethylidene)hydrazono)-2,3-dihydrothiazole-4-carboxylic acid (**1146**). Yield 99% yellow powder, mp: 145–149 °C; ^1^H RMN (400 MHz CDCl_3_) δ (ppm): 0.63 (s, 3H), 0.99 (s, 3H), 2.83 (t, 1H, *J* = 8.5 Hz), 3.52 (m, 1H), 5.35 (m, 3H), 5.21 (d, 1H, *J* = 16Hz), 5.32 (d, 1H, *J* = 10Hz), 5.77 (m, 2H), 7.76 (s, 1H). ^13^C RMN (100 MHz CDCl_3_) δ (ppm): 13.2, 19.4, 21.3, 23.4, 25.6, 31.2, 32.3, 32.6, 37.5, 37.9, 39.2, 42.3, 45.5, 47.4, 50.2, 57.7, 62.3, 71.3, 117.5, 117.9, 121.2, 134.5, 146., 146.9, 158.2, 163.5, 165.8, 180.3. C_28_H_39_N_3_O_3_S. ESI-MS (m/z): 497.27 (100.0%), 498.27 (32.2%), 499.28 (10.2%), 500.27 (1.5%). Elemental analysis: C, 67.57; H, 7.90; N, 8.44; O, 9.64; S, 6.44.


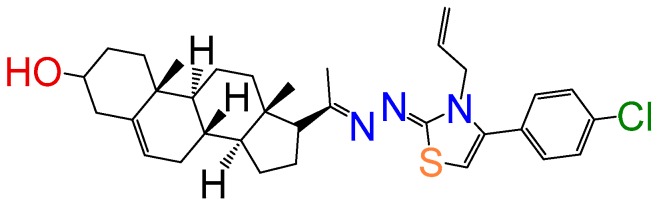


##### Compound **20**

1-(3*β*-Hydroxy-pregn-5-ene-20*E*-ylidene)-2-(3-allyl-4-(4-chloro-pheny)thiazol-2(3*H*)-ylidene)azine (**1144**). Yield 52% yellow powder, mp: 140–144 °C; ^1^H RMN (400 MHz CDCl_3_) δ (ppm): 0.63 (s, 3H), 0.99 (s, 3H), 2.83 (t, 1H, *J* = 8.5 Hz), 3.52 (m, 1H), 5.35 (m, 3H), 5.21 (d, 1H, *J* = 16Hz), 5.32 (d, 1H, *J* = 10Hz), 5.77 (m, 2H), 7.32 (d, 2H, *J* = 10Hz), 7.44 (d, 2H, *J* = 10Hz), 7.76 (s, 1H). ^13^C RMN (100 MHz CDCl_3_) δ (ppm): 13.4, 19.8, 21.3, 23.4, 25.6, 31.3, 32.2, 32.6, 37.5, 37.9, 39.2, 42.4, 45.4, 47.6, 50.2, 57.5, 62.3, 71.3, 117.5, 117.9, 120.2, 121.4, 128.2, 128.5, 133.7, 134.4, 146.6, 146.5, 158.7, 163.5, 165.2. C_33_H_42_ClN_3_OS. ESI-MS (m/z): 563.27 (100.0%), 565.27 (41.9%), 564.28 (38.2%), 566.27 (14.7%), 567.28 (4.4%). Elemental analysis: C, 70.25; H, 7.50; Cl, 6.28; N, 7.45; O, 2.84; S, 5.68.


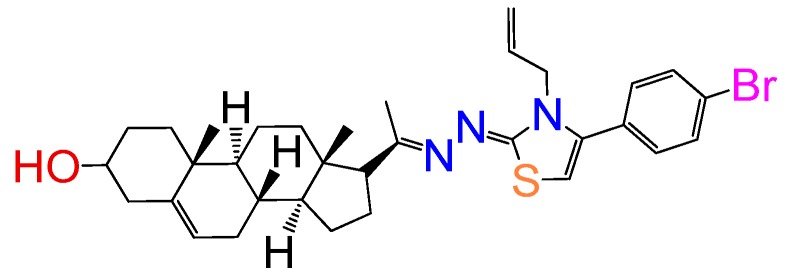


##### Compound **11**

1-(3*β*-Hydroxy-pregn-5-ene-20*E*-ylidene)-2-(3-allyl-4-(4-bromo-pheny)thiazol-2(3*H*)-ylidene)azine (**1272**). Yield 85%, yellow powder, mp: 179–183 °C; ^1^H RMN (400 MHz CDCl_3_) δ (ppm): 0.63 (s, 3H), 0.99 (s, 3H), 2.83 (t, 1H, *J* = 8.5 Hz), 3.52 (m, 1H), 5.35 (m, 3H), 5.21 (d, 1H, *J* = 16Hz), 5.32 (d, 1H, *J* = 10Hz), 5.77 (m, 2H), 6.94 (s, 1H), 7.27 (d, 2H, *J* = 10Hz), 7.55 (d, 2H, *J* = 10Hz). ^13^C RMN (100 MHz CDCl_3_) δ (ppm): 13.2, 19.5, 21.3, 23.4, 25.5, 31.4, 32.5, 32.9, 37.2, 37.8, 39.1, 42.5, 45.6, 47.7, 50.2, 57.5, 62.2, 71.5, 117.2, 117.6, 120.1, 121.5, 128.2, 128.8, 133.2, 134.4, 146.5, 146.5, 158.4, 163.2, 165.5 C_30_H_38_BrN_3_OS. ESI-MS (m/z): 609.22 (100.0%), 607.22 (97.8%), 610.22 (38.4%), 608.23 (36.4%), 609.23 (6.7%), 611.23 (11.6%), 612.22 (1.6%). Elemental analysis: C, 65.12; H, 6.96; Br, 13.13; N, 6.90; O, 2.63; S, 5.27.

#### 3.1.4. General Procedure for the Preparation of Amides

A mixture of the corresponding carboxylic acid (1.0 eq.), carbonyl diimidazole (2.0 eq.), and dry THF (1 mL per 100 mg of acid) was stirred at room temperature for 2 h. After that, the reaction mixture was cooled at 0 °C and the corresponding amine (2.0 eq.) and triethylamine (1.0 eq.) were added. The mixture was stirred at room temperature until the disappearance of the activated acid (12–24 h, checked by TLC, Al_2_O_3_, petroleum ether:EtOAc 70:30). After that, the solvent was evaporated in vacuo and the residue was partitioned between methylene dichloride and saturated aqueous solution of sodium bicarbonate. The organic layer was washed with aqueous phosphate buffer (pH 4–5), dried with anhydrous sodium sulfate, and evaporated in vacuo. The desired product was purified from the residue of evaporation by column chromatography (Al_2_O_3_, petroleum ether:EtOAc 0% to 40%) [26].


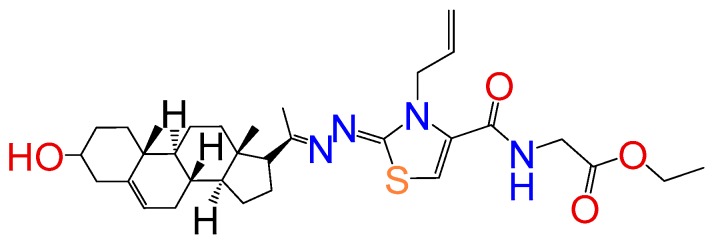


##### Compound **14**

Ethyl-2-((*Z*)-3-allyl-2-((*E*)-(1-((8*S*,9*S*,10*R*,13*S*,14*S*,17*R*)-3-hydroxy-10,13-dimethyl-2,3,4,7,8,9,10,11,12,13,14,15,16,17-tetradecahydro-1*H*-cyclopenta[a]phenanthren-17-yl)ethylidene)hydrazono)-2,3-dihydrothiazole-4-carboxamido)acetate (**1263**). Yield 30%, orange oil. ^1^HRMN (400 MHz CDCl_3_) δ (ppm): 0.63 (s, 3H), 0.99 (s, 3H), 1.29 (m, 3H), 2.83 (t, 1H, *J* = 8.5 Hz), 3.52 (m, 1H), 4.13 (m, 2H), 4.23 (s, 2H), 5.35 (m, 3H), 5.21 (d, 1H, *J* = 16Hz), 5.32 (d, 1H, *J* = 10Hz), 5.77 (m, 2H), 7.76 (s, 1H), 8.03 (s, 1H). ^13^C RMN (100 MHz CDCl_3_) δ (ppm): 13.4, 14.5, 19.6, 21.5, 23.5, 25.4, 31.3, 32.5, 32.9, 37.2, 37.8, 39.2, 40.2, 42.4, 45.5, 47.4, 50.2, 57.7, 62.2, 66.4, 71.2, 117.5, 117.8, 121.4, 134.4, 146.2, 146.2, 158.4, 163.2, 165.3, 169.2. C_32_H_46_N_4_O_4_S. ESI-MS (m/z): 582.32 (100.0%), 583.33 (37.3%), 584.33 (12.2%), 585.32 (2.6%). Elemental analysis: C, 65.95; H, 7.96; N, 9.61; O, 10.98; S, 5.50.


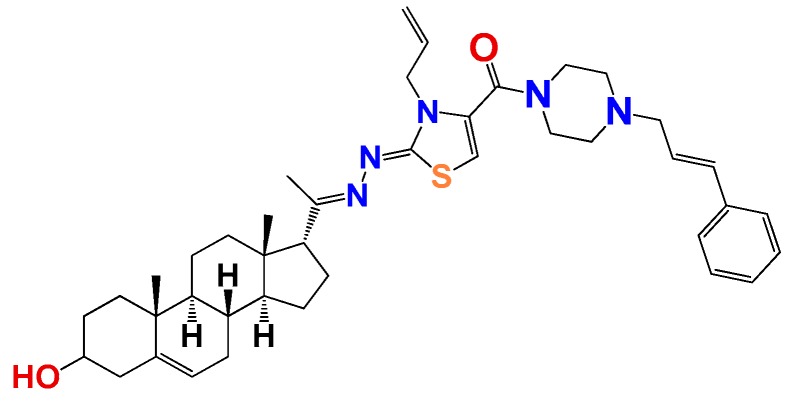


##### Compound **12**

((Z)-3-allyl-2-((E)-(1-((8S,9S,10R,13S,14S,17R)-3-hydroxy-10,13-dimethyl-2,3,4,7,8,9,10,11,12,13,14,15,16,17-tetradecahydro-1H-cyclopenta[a]phenanthren-17-yl)ethylidene)hydrazono)-2,3-dihydrothiazol-4-yl)(4-cinnamylpiperazin-1-yl)methanone (**1261**). Yield 21%, orange oil. ^1^H RMN (400 MHz CDCl_3_) δ (ppm): 0.63 (s, 3H), 0.99 (s, 3H), 2.83 (t, 1H, *J* = 8.5 Hz), 3.52 (m, 1H), 2.79 (dd, 4H, *J* = 1.6 y 4 Hz), 3.02 (s, 2H), 3.33 (dd, 4H, *J* = 1.6 y 4 Hz), 5.35 (m, 3H), 5.21 (d, 1H, *J* = 16 Hz), 5.32 (d, 1H, *J* = 10 Hz), 5.77 (m, 2H), 6.19 (m, 1H), 6.56 (m, 1H), 7.24 (m, 2H), 7.33 (m, 1H), 7.41 (m, 2H), 7.76 (s, 1H). ^13^C RMN (100 MHz CDCl_3_) δ (ppm): 13.2, 193, 21.2, 23.3, 25.5, 31.3, 32.2, 32.6, 37.2, 37.8, 39.2, 42.3, 45.5, 47.5, 50.2, 50.8, 55.1, 57.2, 62.4, 71.4, 117.2, 117.5, 121.3, 127.7, 128.2, 134.2, 136.5, 146.5, 146.9, 158.2, 163.5, 165.5, 180.3 C_40_H_55_N_5_O_2_S. ESI-MS (m/z): 681.41 (100.0%), 682.41 (47.9%), 683.41 (15.7%), 684.41 (4.3%). Elemental analysis: C, 72.21; H, 8.13; N, 10.27; O, 4.69; S, 4.70.


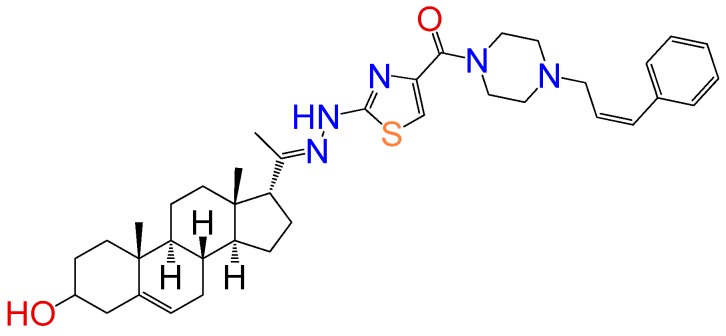


##### Compound **13**

(2-((*E*)-2-(1-((8*S*,9*S*,10*R*,13*S*,14*S*,17*R*)-3-hydroxy-10,13-dimethyl-2,3,4,7,8,9,10,11,12,13,14,15,16,17-tetradecahydro-1*H*-cyclopenta[a]phenanthren-17-yl)ethylidene)hydrazinyl)thiazol-4-yl)(4-((*Z*)-3-phenylallyl)piperazin-1-yl)methanone (**1317**). Yield 15%, orange oil, ^1^H RMN (400 MHz CDCl_3_) δ (ppm): 0.63 (s, 3H), 0.99 (s, 3H), 2.83 (t, 1H, *J* = 8.5 Hz), 3.52 (m, 1H), 2.79 (dd, 4H, *J* = 1.6 y 4 Hz), 3.02 (s, 2H), 3.33 (dd, 4H, *J* = 1.6 y 4 Hz), 5.35 (m, 3H), 6.19 (m, 1H), 6.56 (m, 1H), 7.24 (m, 2H), 7.33 (m, 1H), 7.41 (m, 2H), 7.76 (s, 1H). ^13^C RMN (100 MHz CDCl_3_) δ (ppm): 13.3, 19.5, 21.3, 23.6, 25.2, 31.2, 32.2, 32.9, 37.5, 37.9, 39.2, 42.4, 45.5, 47.5, 50.2, 50.8, 55.2, 57.2, 62.6, 71.2, 117.2, 117.6, 121.2, 127.2, 128.8, 134.4, 136.5, 146.1, 146.5, 158.2, 163.2, 165.6, 180.8 C_40_H_55_N_5_O_2_S. ESI-MS (m/z): 641.38 (100.0%), 682.41 (47.9%), 683.41 (15.2%), 684.41 (5.9%). Elemental analysis: C, 72.21; H, 8.13; N, 10.27; O, 4.69; S, 4.70.

### 3.2. Anti-Parasitic Test In Vitro

*L. amazonensis* and *L. infantum* (MHOM/BR/2002/LPC-RPV) were obtained from Fiocruz (Collection of Oswaldo Cruz Foundation, Rio de Janeiro, Brazil), and promastigotes were cultured as described with some modifications [33,34], at 28 °C in an RPMI medium supplemented with 0.7% glucose, 0.1% ornithine, 0.4% fructose, 0.6% malate, 0.05% fumarate, and 0.06% succinate, 20% Fetal bovine serum, vitamins, and amino acids solution (Gibco). Briefly, all solids (for 1 L) including RPMI were dissolved in 600 mL of distilled water and afterward amino acids and vitamin solutions were added, pH was adjusted to 7.2 with NaOH. Finally, water is added to 800 mL final volume, sterilized by filtration with 0.22 µm pore filter and stored at 4 °C until use (the same culture media described in the next section with *T. cruzi* epimastigotes could be used as a low-cost alternative for *Leishmania spp*. culture). Assays were performed using 2 × 10^6^ promastigotes per well cultivated in 96-well plastic plates. Compounds were dissolved in dimethylsulfoxide (DMSO). Different serial dilutions (25, 12.5, 6.25, 3.13, 1.6, 0.8, 0.4, 0.2, 0.1, 0.05) µM of the compounds with a final volume up to 200 µL were added. After 48 h at 26 °C, 20 µL of a 2 mM resazurin solution was added, and the oxidation-reduction was quantified at 570 and 600 nm. The solution of resazurin was prepared at 2.5 mM in phosphate-buffered solution (PBS), pH 7.4, and filtered through 0.22 µm membranes prior to use. Resazurin sodium salt was obtained from Sigma-Aldrich (St. Louis, MO, USA) and stored at 4 °C protected from light. The efficacy of each compound was estimated by calculating the IC_50_ values using OriginLab8.5^®^ sigmoidal regression. Each anti-proliferative experiment was done in duplicate, and each concentration was tested in triplicate.

For the in vitro anti-*T. cruzi* activity, we used epimastigotes of the Tulahuen 2 strain (genotype TcVI) grown in an axenic milieu (BHI-Tryptose). Cells from a 5–7-days-old culture were inoculated in fresh culture milieu to give an initial concentration of 10^6^ cells/mL. The absorbance at 600 nm of the cells in culture was measured every day. At day five, the milieu was inoculated with different doses of the compounds (25, 12.5, 6.25, 3.13, 1.6, 0.8, 0.4, 0.2, 0.1, 0.05) µM from a stock solution in DMSO (DMSO concentration in the culture milieu never exceeded 0.4%). Control parasites were cultivated in 0.4% DMSO. Each concentration of the compound was evaluated in duplicate. At five days, the absorbance of the culture was measured and compared to the control and the IC_50_ values calculated for each compound using OriginLab8.5^®^ sigmoidal regression. Each experiment was done in duplicate, and each concentration was tested in triplicate.

For assessing the amastigote form of all *Leishmania* strains were cultured in the RPMI described above. Parasites were seeded at 1 × 10^6^ cells/mL and at each time point counted at least five times to obtain a growth curve. For IC_50_ experiments, parasites were seeded at 3 × 10^6^ cells/mL and incubated with serial dilutions of compounds starting from 100 µg/mL Amphotericin B, 50 µM nifurtimox, 25 µM miltefosine. Control conditions of parasites without drug (100% growth) and medium without parasites were included. After 72 h at 28 °C parasite viability was determined by the resazurin method described [35]. Infection with *L. infantum*. THP-1 monocytes (ATCC^®^TIB-202™) were grown following ATCC recommendations and seeded at 30,000 cells/well onto 18-mm round glass coverslips in 12 wells. For stimulation cells were incubated with 100 nM of PMA for 48 h, PMA was washed and cells left with growth media for 24 h more after incubation with parasites. 30,000 parasites/well were added and left to interact. After 48 h, coverslips were washed with PBS, fixed with 95% (*v*/*v*) ethanol, and stained with Fluoroshield™ with DAPI (Sigma). Infectivity was assessed considering invasion and replication capacity counting infected cells and parasites per infected cell, respectively. Infectivity index calculation = (% of infected cells X (amastigotes per cell))/number of total counted cells. Image acquisition was made with a Leica TCS-SP5 laser scanning confocal microscope (Leica Microsystems GmbH, Wetzlar, Germany) using the LASAF v.2 software (Leica Microsystems, Wetzlar, Germany) and observed using a 356 nm laser excitation line and an emission bandwidth from 461 nm under a 20× water immersion objective. Further image analysis, including quantitation and processing of figures were made using Fiji (http://fiji.sc/) [35].

### 3.3. Nonspecific In Vitro Cytotoxicity of Mammalian Cells 

J774.1 murine macrophages (ATCC, USA) were grown in a DMEM culture milieu containing 4 mM L-glutamine and supplemented with 10% FCS. Cells were seeded in a 96-well plate (5.00 × 10^4^ cells in 200 µL culture medium) and incubated at 37 °C in a 5% CO_2_ atmosphere for 48 h, to allow cell adhesion prior to drug testing. Afterward, cells were exposed for 48 h to the compounds (25–400 μM) or the vehicle for control (0.4% DMSO), and additional controls (cells in medium) were used in each test. Cell viability was then assessed by measuring the mitochondria-dependent reduction of MTT (3-(4,5-dimethylthiazol-2-yl)-2,5-diphenyltetrazolium bromide) to formazan. For this purpose, MTT in sterile PBS (0.2% glucose), pH 7.4, was added to the macrophages to achieve a final concentration of 0.1 mg/mL, and the cells were incubated at 37 °C for 3 h. After removing the medium, formazan crystals were dissolved in 180 μL of DMSO and 20 μL of MTT buffer (0.1 M glycine, 0.1 M NaCl, 0.5 mM EDTA, pH 10.5), and the absorbance at 560 nm was measured. The IC_50_ was defined as the drug concentration at which 50% of the cells were viable, relative to the control (no drug added), and was determined by analysis using OriginLab8.5^®^ sigmoidal regression (% of viable cells compared to the logarithm of the compound concentration). Tests were performed in triplicate. For the primary culture of mice macrophages, were taken from the mice bones with PBS, then resuspended in an RPMI culture media, *10^6^ cells per well, incubated immediately with the compound (25 µM), then at 48 h of incubation in an MTT assay was performed [26,33]. 

### 3.4. Vehicles/Formulation Preparation

Compound **8** was disposed in a mixture composed of a surfactant (10%), containing Eumulgin HRE 40 (polyoxyl-40hydrogenated castor oil), sodium oleate, and soya phosphatidylcholine (8:6:3), and an oil phase (10%) containing cholesterol and phosphate buffer (pH 7.4) (80%). For formulation, cholesterol, Eumulgin HRE 40, and phosphatidylcholine previously pulverized in mortar were dissolved in chloroform and the solvent was evaporated under vacuum to dryness. In parallel, sodium oleate was dissolved in phosphate buffer and left in an orbital shaker for 12 h at room temperature. The latter was then added to the evaporated residue, and the mixture was homogenized and placed in an ultrasonic bath at full power for 30 min and kept at room temperature until use [26]. 

### 3.5. In Vivo Micronucleus Test

For the in vivo micronucleus test, approximately three-month-old CD-1 male mice were housed in polycarbonate cages at RT (25 °C) and a photoperiod of 12 h throughout the study. Compound **8** and vehicle were orally administered twice, at days one and two, to groups of five mice at a dose of 150 mg/kg of body weight. Mice were sacrificed 24 h after the last administration, and the bone marrow was prepared for evaluation with slight modifications of the method reported by Schmid [36]. At least two slides of the cell suspension per animal were made. The air-dried slides were stained with Giemsa stain (5% in phosphate buffer, pH 7.4) and examined at 1000x magnification. Small round or oval bodies, the size of which ranged from about 1/5 to 1/20 of the diameter of a polychromatic erythrocyte (PCE), were counted as micronuclei. A total of 1000 PCEs were scored per animal by the same observer for determining the frequencies of micronucleated polychromatic erythrocytes (MNPCEs). Cyclophosphamide, 50 mg/kg, administered intraperitoneally (i.p.) 24 h before mouse sacrifice, was used as a positive control. For statistical analysis, the homogeneity of variances of data was tested by the analysis of variance (ANOVA) test (*P* < 0.05) using the EpiInfo (3.5.1) software [25].

### 3.6. In Vivo Acute Oral Toxicity in Mice 

The in vivo 50% lethal dose (LD_50_) for compound **8** was determined according to the guidelines of the Organization for Economic Cooperation and Development (OECD). Briefly, healthy young adult male B6D2F1 mice (30 days old, 25 to 30 g) were used in this study. Initially, the compound was dissolved in the vehicle described above (3.4 section) and was administered at 2000 mg/kg, by orogastric cannula, to one animal. The animal was fasted, maintained, and observed for 48 h. If the mouse survives, another animal receives the same dose, and 48 h later, a third animal. If there are no toxicity signs the experiment is finished 14 days after administration, with the euthanasia of the animals according to the OECD guidelines. Observations of the general status of the organs were performed after sacrifice. The PROTOX software was used to predict the LD_50_ of compound **8** (http://tox.charite.de/protox_II/) [26]. 

### 3.7. In Vivo Anti-T. Cruzi Studies (Acute Model)

BALB/c male mice (30 days old, 25 to 30 g) bred under specific pathogen-free conditions were infected by intraperitoneal injection with 1x10^3^ blood trypomastigotes of the Y strain. The mice were divided into three groups. One group of animals, *n* = 8, was used as a control (treated orally with the vehicle), and two groups of animals, *n* = 8 each, were treated with compound **8** and Bnz. Initial parasitemia was counted five days postinfection (Week 1), and the treatment was begun the following day (6th day). Bnz (at 50 mg/kg body weight [BW]/day, for 14 days) or **8** (at 50 mg/kg BW/day, for 14 days) were administered orally, using the described vehicle. Parasitemia in the control and treated mice were determined once a week after the first administration, for 60 days after the beginning of treatment, in tail vein blood. Additionally, the mortality rate was recorded [26].

### 3.8. In Vivo Anti-Leishmania Studies in Cutaneous Mice Model

Female and male BALB/c mice were supplied by the IFFA-CREDO, Lyon, France, and were bred at the Instituto de Investigaciones en Ciencias de la Salud, Asuncion, Paraguay. Golden hamsters (*Mesocritus auratus*) were used to maintain the parasites. For the infection *L. amazonensis* (IFLA/BR/1967/PH8) were used. The parasites were maintained by passage every six to eight weeks in hamsters. BALB/c mice (n = 8) were inoculated in the right hind footpad with 2 × 10^6^ amastigotes obtained from donor hamsters. The parasites were delivered in 100 µL of phosphate-buffered saline (PBS). Disease progression was monitored by the measurement of lesion diameters weekly for up to seven to 12 weeks. In all experiments, treatment was initiated one or two weeks after inoculation, when the infection was well established and lesions were obvious. Two days before the administration of the drug, the mice were randomly divided into groups of eight. *N*-Methylglucamine antimonate was administered to the BALB/c mice 100 mg/Kg of body weight daily for 20 days by the subcutaneous route. Compound **8** was administered orally at 50 mg/Kg of bodyweight. The animals were sacrificed two weeks after the cessation of treatments to assess parasitological loads in the infected footpad. Briefly, the mice were sacrificed, and the lesions of the infected footpad were excised, weighed, and homogenized in a tissue glass grinder and then homogenized in 5 µl of RPMI 1640 (GIBCO, Paris, France) tissue culture medium supplemented with 10% FCS, 1 mL of glutamine (29.4 µg/mL; GIBCO), penicillin (100 U/mL), and streptomycin (100 µg/mL). After seven days of incubation at 27 °C, the plates (25 cm^2^ Falcon T) were examined with an inverted microscope (Olympus) at a magnification of 400x. The number of parasites per gram in the lesion was calculated by the following equation: Parasite burden = geometric mean of the number of parasites in each duplicate/(number of microscope field counted x weight of lesion x (25000) hemocytometer correction factor). Statistical analysis: The mean and standard deviation were calculated by using Microsoft EXCEL software. Comparisons of parasite suppression in the infected footpads of the untreated and drug-treated groups were done by analysis of variance (ANOVA) and Student’s T-test. Data were considered statistically significant at *P* < 0.05 [37].

### 3.9. Calculation of the Pharmacokinetic Parameters

The predictions were made with the open-access SwissADME software (http://www.swissadme.ch), a tool that allows the prediction of different pharmacokinetic parameters such as water solubility, gastrointestinal absorption, skin penetrability, lipophilicity, bioavailability, etc (Figure S1). The SwissADME software input uses the SMILES codes of the molecules, which were generated with the ChemBioOffice 2010 program [38].

### 3.10. Liver fraction Stability Studies

For the determination of liver fraction stability, rat liver microsomal and cytosolic proteins were used. They were prepared according to the previously described methodology [39]. The protein content of the microsomal and cytosolic fractions was determined by the bicinchoninic acid assay from Sigma, as suggested by the manufacturer. The final concentration in the aqueous medium of **8** was 400 µM and was prepared from a stock solution in DMSO. The solutions were further homogenized and incubated at 37 °C for 1 to 4 h. After that, thin-layer chromatography of ethyl acetate extracts was performed in order to evaluate the presence of decomposition products (Figure S2).

## 4. Conclusions

In this work, we introduced a new class of steroids as anti-kinetoplastid agents. We evaluated the trypanosomicidal effect in *T. cruzi* and *Leishmania spp.* in vitro and in vivo, and also studied the toxicological profile of the active compounds. Nineteen new compounds were synthesized and characterized, six of them demonstrating antiparasitic activity. The most active derivative, compound **8** has similar or better in vitro and in vivo activity compared to the drugs Benznidazole, Glucantime, and Miltefosine currently used for the treatment of these diseases. This compound has a good toxicological profile, high selectivity index, and promising pharmacokinetic parameters since good absorption and distribution in vivo are predicted. These compounds can be orally administered and have a low-cost production, which makes them promising candidates for the development of drugs for the control of neglected diseases. 

## Figures and Tables

**Table 1:** Trypanocidal activity against *T. cruzi* (epimastigotes), *L. amazonensis* (promastigotes) and *L. infantum* (promastigotes).

STRUCTURE	No *	IDENTIFIER ^#^	IC_50_ ± %SD (µM) *T. cruzi*	IC_50_ ± %SD (µM) *L. amazonensis*	IC_50_ ± %SD (µM) *L. infantum*	IC_50_ ± %SD (µM) L. infantum .uy **
		**Nifurtimox^®^**	**7 ± 2**	**Nd ^##^**	**6 ± 2**	**10±2**
		**Glucantime^®^**	**Nd**	**18 ± 2**	**20 ± 9**	**nd**
		**Miltefosine^®^**	**8 ± 2**	**nd**	**0.9 ± 0.2**	**5 ± 2**
		**Pregnenolone**	**˃25**	**˃25**	**˃25**	**˃25**
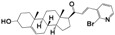	**1**	**1257**	**˃25**	**˃25**	**˃25**	**˃25**
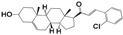	**2**	**1259**	**12 ± 3**	**23 ± 5**	**˃25**	**˃25**
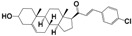	**3**	**1256**	**˃25**	**˃25**	**˃25**	**˃25**
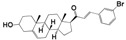	**4**	**1417**	**20 ± 5**	**˃25**	**˃25**	**˃25**
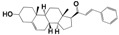	**5**	**1288**	**˃25**	**˃25**	**˃25**	**˃25**
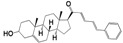	**6**	**1279**	**˃25**	**˃25**	**˃25**	**˃25**
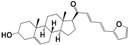	**7**	**1289**	**˃25**	**˃25**	**˃25**	**˃25**
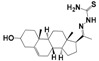	**8**	**1260**	**1.2 ± 0.3**	**<22**	**0.2 ± 0.1**	**0.2 ± 0.1**
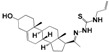	**9**	**1154**	**8 ± 2**	**nd**	**˃25**	**˃25**
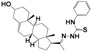	**10**	**1291**	**25 ± 3**	**nd**	**˃25**	**˃25**
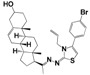	**11**	**1272**	**˃25**	**16 ± 3**	**˃25**	**˃25**
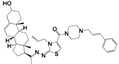	**12**	**1261**	**˃25**	**nd**	**˃25**	**˃25**
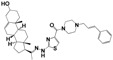	**13**	**1317**	**˃25**	**nd**	**˃25**	**˃25**
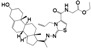	**14**	**1263**	**8 ± 2**	**nd**	**˃25**	**˃25**

* Compounds **15**—**20** were inactive, total data in Table S1 ^#^ Compounds numbers in our chemical collection ** parasites isolated from two dogs in the first Leishmaniasis outbreak in Uruguay [29]. ^##^ no determined.

**Table 2:** Nonspecific cytotoxicity test was carried out with J774.1 murine macrophages and selectivity indexes were calculated for *L. amazonensis* (promastigotes), *L. infantum* (promastigotes), and *T. cruzi* (epimastigotes).

Compound	IC_50_ ± SD (µM) Murine Macrophages	SI * J774.1/*L. amazonensis*	SI J774.1/*L. infantum*	SI J774.1/*T. cruzi*
**2**	100 ± 10	4	nd	8
**8**	50 ± 3	>2	250	42
**9**	25 ± 2	nd^#^	nd	3
**10**	25 ± 5	nd	nd	1
**14**	100 ± 8	nd	nd	13
**Glucantime**	15 ± 1	1	0.5	nd
**Miltefosine**	50 ± 7	nd	56	6
**Benznidazole**	400 ± 4	nd	nd	57

* SI Selectivity index is the ratio between IC_50_ in murine macrophages and the IC_50_ in the parasite. ^#^no determined.

**Table 3:** Micronucleus test using 150 mg/Kg of compound **8** in mice.

Treatment *	Number of MnPE **	Number of PEC ^+^	Media of MnPE ±SD ^++^
Control	19	5000	4 ± 1
**8**	24	5000	5 ± 1
Cyclophosphamide *** (50 mg/kg)	180	5000	36 ± 2

* Five identical tests were performed at independent times, ** Sum of the micronucleated polychromatic erythrocytes (MnPE) found in the five trials, ^+^ Total number of polychromatic erythrocytes observed, ^++^ Media of MnPE per mouse ± standard deviation *** by intraperitoneal administration.

**Table 4:** In vivo results for the oral administration of 50 mg/kg of compound **8** in mice models of acute Chagas and Leishmaniasis diseases.

Treatment	Doses mg/kg	Doses µmol/kg	Number of Parasites	% of Reduction	% of Survivals
**Chagas**					
Control	0	0	(0.38 ± 0.02)×10^6^ *	0	60
**8**	50	127	(0.14 ± 0.03) × 10^6^ *	62	100
**Benznidazole**	50	200	(0.01 ± 0.01) × 10^6^ *	96	100
**Leishmaniasis**					
Control	0	0	(29 ± 2) × 10^6^	0	100
**8**	50	127	(21 ± 4) × 10^6^	27	100
**Glucantime**	100	273	(25 ± 6) × 10^6^	12	100

* number of parasites per mL blood in the peak of parasitemia.

**Table 5:** Pharmacokinetic parameters predicted using SwissADME.

Compound	Solubility (mg/mL)	Gastrointestinal Abortion	Brain Permeability	Skin Penetration (cm/s)	Bioavailability Score	Lipophilicity
Miltefosine	1.9 × 10^−3^	low	No	−4.0	0.55	3.8
Glucantime	2.2 × 10^3^	low	No	−11.3	0.55	−2.9
Benznidazole	2.3	high	No	−7.2	0.55	0.5
8	9.7 × 10^−3^	high	No	−5.8	0.55	3.9

## Supplementary Materials

The following are available online at https://www.mdpi.com/1420-3049/24/20/3800/s1, Table S1. Total data from the in vitro assays with the parasites for 20 compounds, Figure S1. Pharmacokinetic r profiles data output from SwissADME, Figure S2 TLC from the hepatic metabolic stability assay in vitro.
