# Supplementary material for: A Nature-Inspired Design Yields a New Class of Steroids Against Trypanosomatids
Source: Molecules. 2019 Oct 22;24(20):3800. doi: 10.3390/molecules24203800 (PMC6832524; doi:10.3390/molecules24203800)
Supplement: Supplementary file 1 [file molecules-24-03800-s001.pdf]

A nature-inspired design yields a new class of steroids against tripanosomatids.

Elena Aguilera[1#], Cintya Perdomo[2#], Alejandra Espindola[2], Ileana Corvo[2], Paula Faral-Tello[3], Carlos Robello[3,4], Elva Serna[5], Fátima Benítez[5,6], Rocío Riveros[5,6], Susana Torres[5], Ninfa I. Vera de Bilbao[5], Gloria Yaluff[5*], Guzmán Alvarez[2*]

[1] Grupo de Química Medicinal-Laboratorio de Química Orgánica, Facultad de Ciencias, Universidad de la República, Montevideo, C.P. 11400, Uruguay; elepao168@gmail.com (E.A.).

[2] Laboratorio de Moléculas Bioactivas, CENUR Litoral Norte, Universidad de la República, Ruta 3 (km 363), Paysandú, C.P. 60000, Uruguay; cuquis266@gmail.com (C.P.), ale.espindola1991@gmail.com (A.E.), ilecorvo@gmail.com (I.C.), guzmanalvarezlqo@gmail.com (G.A.).

[3] Unidad de Biología Molecular, Institut Pasteur de Montevideo, Montevideo, C.P. 11400, Uruguay; pfaral@pasteur.edu.uy (P.F-T), robello@pasteur.edu.uy (C.R.).

[4] Departamento de Bioquímica, Facultad de Medicina, Universidad de la República, Montevideo, Uruguay

[5] Departamento de Medicina Tropical, Instituto de Investigaciones en Ciencias de la Salud, Universidad Nacional de Asunción, San Lorenzo, C.P. 2169., Paraguay; elvsern@gmail.com (E.S.), susitorres1@hotmail.com (S.T.), nverabilbao@gmail.com (N. I. V. de B.), gloriayaluff@yahoo.com (G.Y.).

[6] Facultad de Ciencias Exactas y Naturales, Universidad Nacional de Asunción, San Lorenzo, C.P. 2169., Paraguay; fatimaapodaca14@gmail.com (F.B.), r.riverosmaidana@gmail.com (R.R.).

# Both authors contribute equally to this work.

* Correspondence: GY: gloriayaluff@yahoo.com, GA: guzmanalvarezlqo@gmail.com; Tel.: +59899274984

Table 1S. Total data from the in vitro assays with the parasites for 20 compounds

STRUCTURE	IDENTIFIER	IC_{50} ± %SD (μM) epimastigotes *T. cruzi*	IC_{50} ± %SD (μM) promastigotes *L. braziliensis/ L. amazonensis*	IC_{50}± %SD (μM) promastigote s *L. infantum*	IC_{50}± %SD (μM) promastigote s *L. infantum uy*p/c*
	**Nifurtimox®**	7±2	nd	6±2	10±2[c]
	**Glucantime®**	nd	18±2	20±9	nd
	**Miltefosine®**	8±2	nd	0.9±0.2	5±2
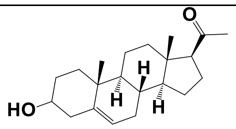	**1054**	>25	>25	>25	>25
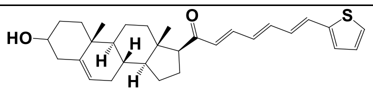	**1319**	>25	>25	>25	>25
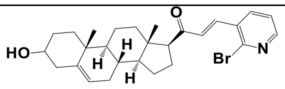	**1257**	>25	>25	>25	>25
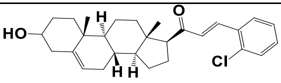	**1259**	12±3	23±5	>25	>25
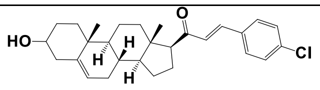	**1256**	>25	>25	>25	>25
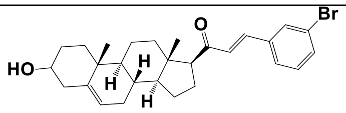	**1417**	20±5	>25	>25	>25
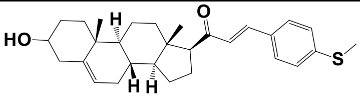	**1262**	>25	>25	>25	>25
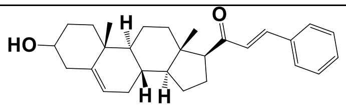	**1288**	>25	>25	>25	>25
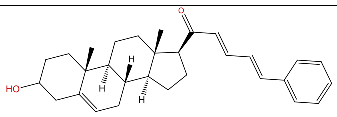	**1279**	>25	>25	>25	>25
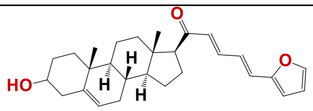	**1289**	>25	>25	>25	>25
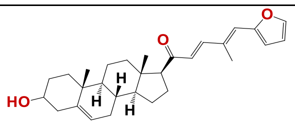	**1287**	>25	23±3	>25	>25
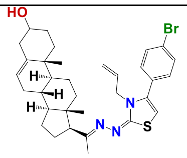	**1272**	>25	16±3	>25	>25

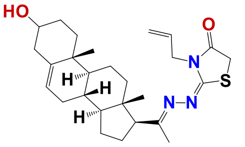	**1125**	>25	<20	>25	25/>25
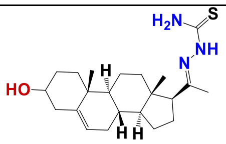	**1260**	1.2	<22	0.2±0.1	0.2
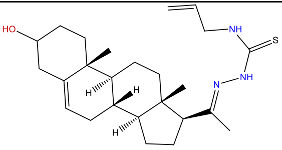	**1154**	7,8	nd	>25	>25
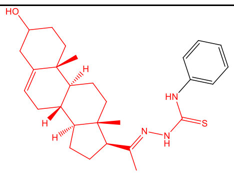	**1291**	25	nd	>25	>25
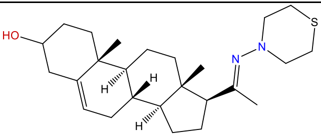	**1087**	>25	>25	>25	nd
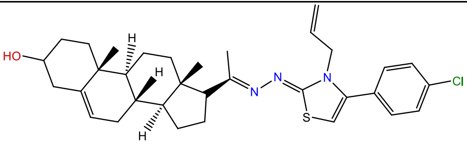	**1144**	>25	>25	>25	nd
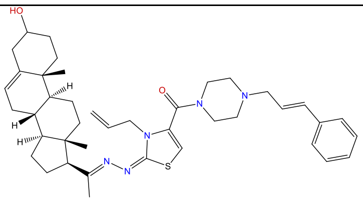	**1261**	>25	>25	>25	>25
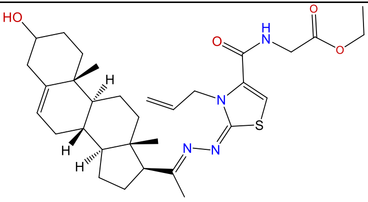	**1263**	8	>25	>25	>25
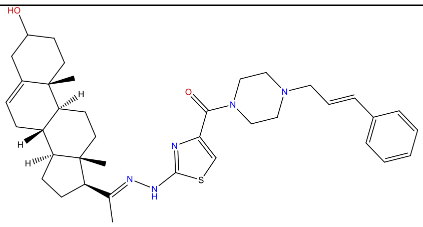	**1317**	>25	>25	>25	>25

Figure 1S. Pharmacokinetic r profiles data output from SwissADME.

A) Glucantime

Molecule 1

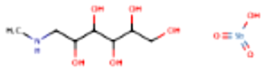

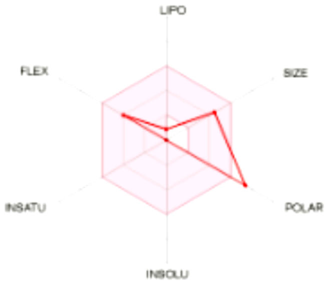

SMILES O[Sb](=O)=O.CNCC(C(C(C(CO)O)O)O)O

Physicochemical Properties	
Formula	C7H18NO8Sb
Molecular weight	365.98 g/mol
Num. heavy atoms	17
Num. arom. heavy atoms	0
Fraction Csp3	1.00
Num. rotatable bonds	6
Num. H-bond acceptors	9
Num. H-bond donors	7
Molar Refractivity	47.68
TPSA	167.55 Å²

Lipophilicity	
Log $P_{o/w}$ (iLOGP)	0.00
Log $P_{o/w}$ (XLOGP3)	-3.96
Log $P_{o/w}$ (WLOGP)	-3.77
Log $P_{o/w}$ (MLOGP)	-5.03
Log $P_{o/w}$ (SILICOS-IT)	-1.72
Consensus Log $P_{o/w}$	-2.90

Water Solubility	
Log *S* (ESOL)	0.78
Solubility	2.21e+03 mg/ml ; 6.05e+00 mol/l
Class	Highly soluble
Log *S* (Ali)	1.04
Solubility	4.01e+03 mg/ml ; 1.10e+01 mol/l
Class	Highly soluble
Log *S* (SILICOS-IT)	1.55
Solubility	1.29e+04 mg/ml ; 3.53e+01 mol/l
Class	Soluble

Pharmacokinetics	
GI absorption	Low
BBB permeant	No
P-gp substrate	No
CYP1A2 inhibitor	No
CYP2C19 inhibitor	No
CYP2C9 inhibitor	No
CYP2D6 inhibitor	No
CYP3A4 inhibitor	No
Log K_p (skin permeation)	-11.34 cm/s

Druglikeness	
Lipinski	Yes; 1 violation: NHorOH>5
Ghose	No; 1 violation: WLOGP<-0.4
Veber	No; 1 violation: TPSA>140
Egan	No; 1 violation: TPSA>131.6
Muegge	No; 3 violations: XLOGP3<-2, TPSA>150, H-don>5
Bioavailability Score	0.55

Medicinal Chemistry	
PAINS	0 alert
Brenk	1 alert: heavy_metal
Leadlikeness	No; 1 violation: MW>350
Synthetic accessibility	3.51

B) Compound 1260

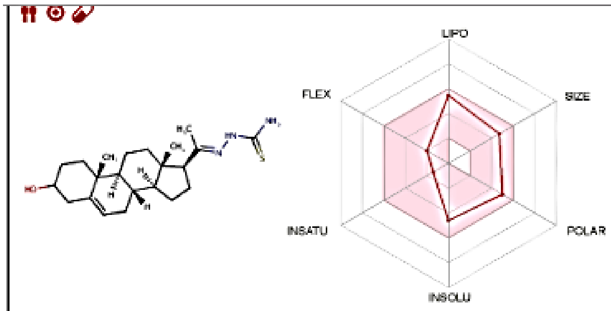

SMILES: OC1CC[C@]2(C(=CC[C@@H]3[C@@H]2CC[C@]2([C@H]3CC[C@@H]2/C(=N/NC(=S)N)/C)C)C1)C

Physicochemical Properties	
Formula	C22H35N3OS
Molecular weight	389.60 g/mol
Num. heavy atoms	27
Num. arom. heavy atoms	0
Fraction Csp3	0.82
Num. rotatable bonds	3
Num. H-bond acceptors	2
Num. H-bond donors	3
Molar Refractivity	116.56
TPSA	102.73 Å²

Lipophilicity	
Log $P_{o/w}$ (iLOGP)	3.57
Log $P_{o/w}$ (XLOGP3)	4.04
Log $P_{o/w}$ (WLOGP)	4.14
Log $P_{o/w}$ (MLOGP)	3.65
Log $P_{o/w}$ (SILICOS-IT)	3.96
Consensus Log $P_{o/w}$	3.87

Water Solubility	
Log *S* (ESOL)	-4.60
Solubility	9.73e-03 mg/ml ; 2.50e-05 mol/l
Class	Moderately soluble
Log *S* (Ali)	-5.90
Solubility	4.90e-04 mg/ml ; 1.26e-06 mol/l
Class	Moderately soluble
Log *S* (SILICOS-IT)	-3.63
Solubility	9.22e-02 mg/ml ; 2.37e-04 mol/l
Class	Soluble

Pharmacokinetics	
GI absorption	High
BBB permeant	No
P-gp substrate	Yes
CYP1A2 inhibitor	No
CYP2C19 inhibitor	Yes
CYP2C9 inhibitor	Yes
CYP2D6 inhibitor	No
CYP3A4 inhibitor	No
Log K_p (skin permeation)	-5.81 cm/s

Druglikeness	
Lipinski	Yes; 0 violation
Ghose	Yes
Veber	Yes
Egan	Yes
Muegge	Yes
Bioavailability Score	0.55

Medicinal Chemistry	
PAINS	0 alert
Brenk	3 alerts: imine_1, isolated_alkene, thiocarbonyl_group
Leadlikeness	No; 2 violations: MW>350, XLOGP3>3.5
Synthetic accessibility	5.84

Figure 2S. TLC from the hepatic metabolic stability assay in vitro. FM (Microsomal fraction) FC (cytosolic Fraction).

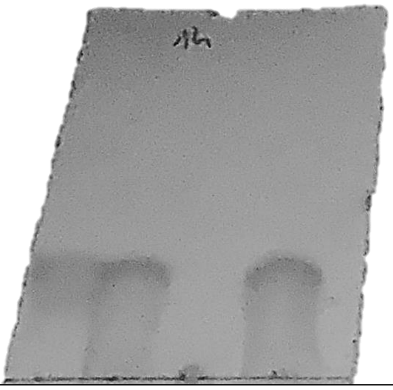

1260 FM FM_only 1260+FM

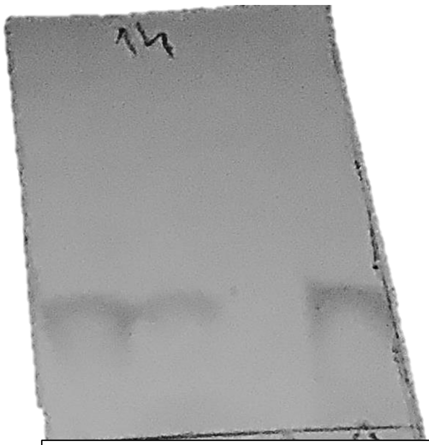

1260 FC FC_only 1260+FM
